# Mosquito Bite and Adnexal Mass: The Unusual Diagnosis of Ovarian Filariasis

**DOI:** 10.7759/cureus.16772

**Published:** 2021-07-31

**Authors:** Mamta R Datta, Mousumi D Ghosh, Vina Kumari, Radhika Narayan

**Affiliations:** 1 Obstetrics and Gynaecology, Tata Main Hospital, Jamshedpur, IND; 2 Pathology, Tata Main Hospital, Jamshedpur, IND

**Keywords:** filaria, ovary, ovarian cyst, tropical disease, mosquito bite

## Abstract

Lymphatic filariasis is a major health problem in tropical regions especially in India. A large number of patients tend to be asymptomatic. Ovarian filariasis is an extremely rare manifestation of lymphatic filariasis. This is a case report of bilateral ovarian filariasis presenting as ovarian mass with associated lower abdominal pain, weight loss and chyluria. This is a very rare diagnosis, more so as it was diagnosed preoperatively by ultrasound and managed with anti-filarial drugs and confirmed by biopsy. Most cases of ovarian filariasis reported in literature are incidental diagnosis on histopathological examination of postoperative specimen.

## Introduction

Lymphatic filariasis is a mosquito-borne parasitic disease caused by round worms of the genera Wuchereria and Brugia. Lymphatic filariasis represents the second leading cause of permanent disability worldwide according to the World Health Organization [1]. Pelvic or ovarian sites are rare and very few cases are reported in literature. Moreover, ovarian filariasis has been rarely diagnosed preoperatively as the condition is commonly confused with a functional cyst [2]. Thus, in most cases reported in literature, the histopathologic examination revealed the incidental diagnosis of ovarian filariasis. Surgery and medical therapy primarily help to avoid recurrences [2].

We report a case of ovarian localization of filariasis in a 26-year-old patient diagnosed before surgery and treated with anti-filarial drugs. The diagnosis was confirmed on histopathology examination of the specimen post-surgery.

## Case presentation

A 26-year-old Indian lady attended outpatient department with history of pain abdomen, weight loss and anorexia for three months and history of chyluria for one month.

She was primipara with last childbirth 14 months back. Her menstrual cycles were irregular, at 40-45 days interval with 5-6 days of bleeding. She was extremely thin built with a BMI of 16.2 at the time of examination. Per abdominal examination was normal. On per vaginal examination, uterus was normal in size. A firm mass was felt in the anterior fornix just above the uterus.

**Figure 1:**
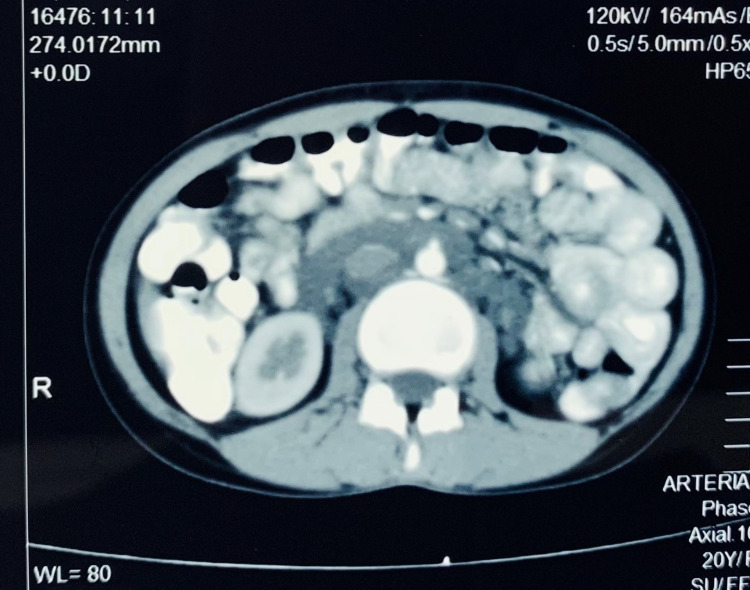
Axial section image of contrast-enhanced computerized tomography of the abdomen showing hypodense non-enhancing lesions in the retroperitoneum surrounding the aorta and the inferior vena cava.

Ultrasound showed complex multi-loculated cystic lesion in pelvis and bilateral adnexa of size 9.1 x 6.5 cm on right side and 4.5 x 6.5 cm on left side. The cystic areas within the mass showed classical ‘microfilariae dance’ or movement of microfilaria. A well-defined echogenic lesion was seen in left ovary of size 3.2 x 3.7 cm suggestive of dermoid cyst. Contrast enhanced computerized tomography (CECT) showed bilateral enlarged cystic ovaries with a dermoid cyst on the left with retroperitoneal lymphadenopathy (Figure 1).

**Figure 2:**
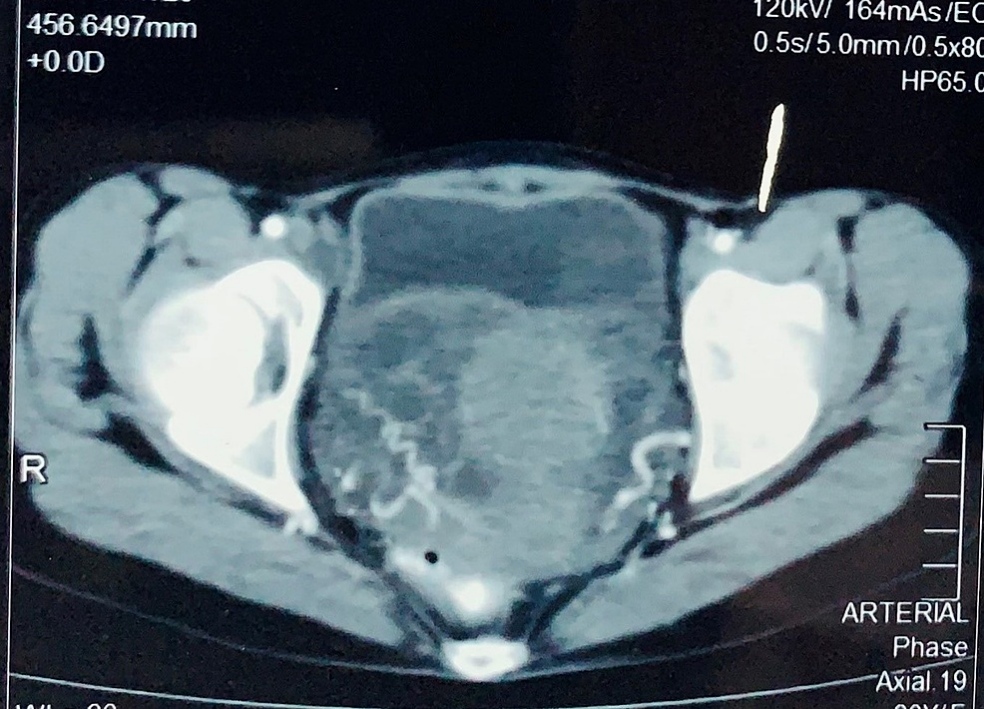
Axial section image of contrast-enhanced computerized tomography of the pelvis showing bilateral adnexal lobulated multicystic non-enhancing masses.

The scan also showed hypodense non-enhancing lesions surrounding the aorta, inferior vena cava and the renal vessels (Figure 2). Multiple mildly enlarged para-aortic, para-caval and aortocaval nodes were seen. Right ovary measured 8.7 x 4.7 cm and the left ovary measured 5.2 x 4.2 cm on CT scan.

The differential diagnoses considered were ovarian filariasis with dermoid cyst, tubercular tubo-ovarian mass or a malignant ovarian mass. Filariasis was confirmed by filarial antigen test positivity in blood. Mantoux test was negative. Tumor markers for ovarian malignancy were negative.

She was put on diethylcarbamazine citrate (DEC) 100 mg thrice daily and advised to return for follow-up after one month. Once on DEC, her general condition improved. Her chyluria was relieved.

**Figure 3:**
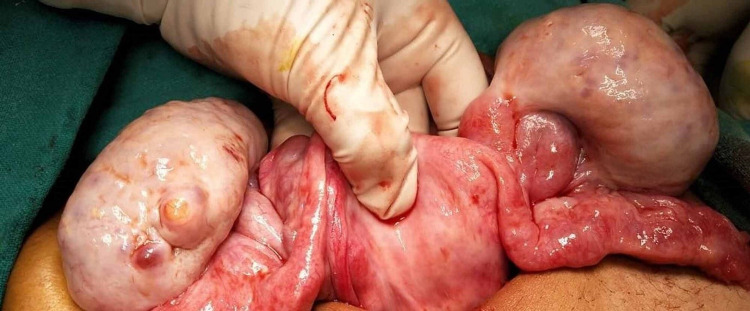
Uterus and ovaries as seen at laparotomy, after medical treatment of filariasis.

Ultrasound was repeated after one month of starting DEC. As the ‘micro-filarial dance’ was still visible on ultrasonography, DEC was continued. Repeat ultrasound after three weeks showed absence of ‘micro-filarial dance’. She was then taken up for laparotomy. On laparotomy, both ovaries were enlarged and fleshy (Figure 3). Left ovary measured approximately 12 x 8 x 6 cm and right ovary measured approximately 10 x 7 x 6 cm. Both tubes were normal. Bilateral ovarian biopsies were taken. The dermoid cyst on the left side was removed. Another small cyst 2.5 cm in diameter with cheesy contents embedded in the right ovary was also removed. The postoperative period was uneventful. Follow-up visits at six weeks and three months after surgery showed that the patient had gained weight and had good appetite and sense of wellbeing. Follow-up ultrasound after three months of surgery showed normal uterus and ovaries.

**Figure 4:**
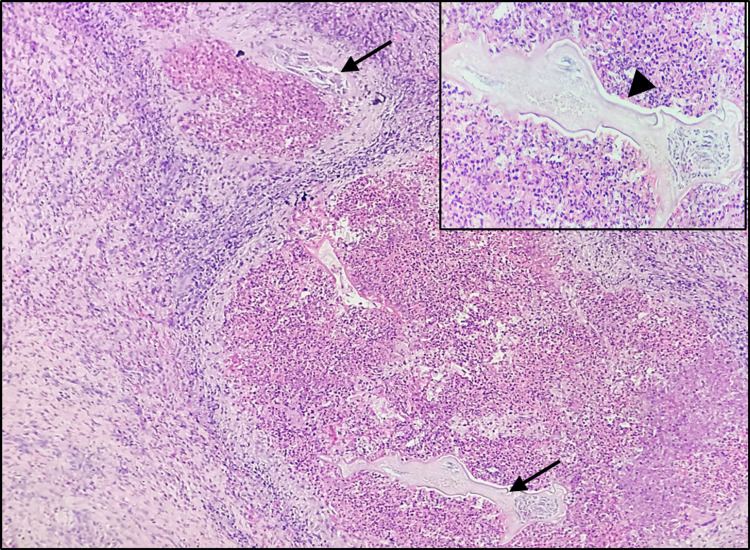
Section of ovary showing two foci of Filarial worms (black arrows) surrounded by dense mixed inflammatory reaction, H&E x100. Inset – higher magnification of the filarial worm (black arrow head), H&E x400.

The histopathology report of right ovarian tissue showed focal presence of microfilariae larvae in the ovarian stroma, surrounded by dense mixed inflammation, predominantly eosinophils (Figure 4). Left ovarian cyst showed features of mature cystic teratoma with focal sections of microfilaria larvae and foreign body giant cells.

## Discussion

Malaria and lymphatic filariasis are two of the most common mosquito-borne parasitic diseases worldwide which can occur as concomitant human infections while also sharing common mosquito vectors [3]. Lymphatic filariasis is a parasitic disease caused by worms that appear thread like under the microscope. The adult worms live in the human lymphatic system. The disease is borne from person to person by infected mosquito bites [4]. India is an endemic zone for lymphatic filariasis. Infection is more common in men than in women. Filariasis causes elephantiasis, lymphedema and in men, it causes hydrocele [3,5]. It can also cause swelling of breasts and genitals [4-5]. The ovarian localization of lymphatic filariasis is rare and only a few cases have been reported in the literature [3].

These parasites are grouped as nematodes (roundworms) of the family Filarioidea. Most infections worldwide are caused by Wuchereria bancrofti. In Asia, the disease can also be caused by Brugia malayi and Brugia timori [4].

Sethi et al. reported two cases of filariasis, one in the ovary and the other in the mesosalpinx. In the first case, the patient underwent pan-hysterectomy and in the second case, right ovarian cystectomy with right salpingectomy was performed. Both patients presented with complaints related to gynecological problems and not filariasis [6]. The diagnosis in these cases was made post-operatively. Mondal et al. reported a case of filarial worm affecting right ovary in a 35‑year‑old female patient who presented with chronic pelvic pain [7]. They performed right-sided salpingo-oophorectomy for tubo-ovarian mass detected on ultrasonography in their patient. The diagnosis of ovarian filariasis was made on histopathologic examination of the ovary [7]. Physicians practicing in non-endemic areas rarely consider filariasis [8].

Additionally, the diagnosis of ovarian filariasis is rarely made in preoperative condition because clinical symptomatology simulates that of a functional cyst [2]. Thus, in most cases reported in literature, the histologic examination revealed the incidental diagnosis of ovarian filariasis.

This is a rare case report because the diagnosis was made pre-operatively based on the clinical picture and radiological investigations. The patient received anti-filarial drugs and the micro-filarial dance seen on ultrasound disappeared after around two months of specific therapy. The diagnosis was finally confirmed by histopathological examination.

Surgery and medical therapy primarily help to avoid recurrences [1,5]. The prognosis of this disease is usually favorable with treatment [2].

## Conclusions

We conclude that ovarian filariasis is a possibility in endemic areas. The diagnosis must be considered in the differential diagnosis of tubo-ovarian or ovarian masses. This can be diagnosed radiologically and easily treated with anti-filarial drugs. Histopathological report confirms the diagnosis. The patients recover and have good prognosis.
